# Nod2 Suppresses *Borrelia burgdorferi* Mediated Murine Lyme Arthritis and Carditis through the Induction of Tolerance

**DOI:** 10.1371/journal.pone.0017414

**Published:** 2011-02-28

**Authors:** Tanja Petnicki-Ocwieja, Alicia S. DeFrancesco, Erin Chung, Courtney T. Darcy, Roderick T. Bronson, Koichi S. Kobayashi, Linden T. Hu

**Affiliations:** 1 Division of Geographic Medicine and Infectious Diseases, Tufts Medical Center, Boston, Massachusetts, United States of America; 2 Department of Pathology, Harvard Medical School, Boston, Massachusetts, United States of America; 3 Cancer Immunology and AIDS, Dana-Farber Cancer Institute, Harvard Medical School, Boston, Massachusetts, United States of America; Academic Medical Center, Netherlands

## Abstract

The internalization of *Borrelia burgdorferi*, the causative agent of Lyme disease, by phagocytes is essential for an effective activation of the immune response to this pathogen. The intracellular, cytosolic receptor Nod2 has been shown to play varying roles in either enhancing or attenuating inflammation in response to different infectious agents. We examined the role of Nod2 in responses to *B. burgdorferi*. *In vitro* stimulation of Nod2 deficient bone marrow derived macrophages (BMDM) resulted in decreased induction of multiple cytokines, interferons and interferon regulated genes compared with wild-type cells. However, *B. burgdorferi* infection of Nod2 deficient mice resulted in increased rather than decreased arthritis and carditis compared to control mice. We explored multiple potential mechanisms for the paradoxical response in *in vivo* versus *in vitro* systems and found that prolonged stimulation with a Nod2 ligand, muramyl dipeptide (MDP), resulted in tolerance to stimulation by *B. burgdorferi*. This tolerance was lost with stimulation of Nod2 deficient cells that cannot respond to MDP. Cytokine patterns in the tolerance model closely paralleled cytokine profiles in infected Nod2 deficient mice. We propose a model where Nod2 has an enhancing role in activating inflammation in early infection, but moderates inflammation after prolonged exposure to the organism through induction of tolerance.

## Introduction

Lyme disease is an infection caused by a tick-borne spirochete, *Borrelia burgdorferi* [1]–[3]. It is the most common vector-borne disease in the United States with 29,959 definite cases and 8,509 probable cases reported in the U.S. in 2009 according to the Centers for Disease Control and Prevention. The clinical manifestations of Lyme disease most typically begin with a characteristic skin lesion, erythema migrans, which originates at the site of the tick bite [1]. Over days to weeks, the organism spreads from the site of the original tick bite to infect distant skin sites, the nervous system, the heart and joints. *B. burgdorferi* causes both acute and chronic infection, as bacterial infection persists even after the resolution of symptoms. Arthritis manifests predominantly in the larger weight-bearing joints and is classified as chronic inflammatory arthritis.

*B. burgdorferi* induces early host pro-inflammatory responses through the activation of Toll-like receptors (TLRs). Of the TLRs, TLR2 appears to be primarily responsible for induction of inflammatory modulators in response to *B. burgdorferi*, which expresses a high number of lipoproteins [4], [5]. Phagocytosis of *B. burgdorferi* is required for the complete activation of the inflammatory response [6]–[9], and several intracellular pathogen recognition molecules, including nucleotide-binding oligomerization domain containing 2 (Nod2), have been shown to play a role in transducing signals downstream of acute *B. burgdorferi* infection *in vitro* [10]–[12]. However, the role of Nod2 has not previously been examined in an *in vivo* model of *B. burgdorferi* infection.

Murine infection with *B. burgdorferi* results in the development of arthritis and carditis in susceptible strains of mice. Mice with *B. burgdorferi* induced arthritis or carditis show increases in multiple inflammatory mediators including tumor necrosis factor alpha (TNF-α), interleukin 6 (IL-6) and interferon regulated genes such as, *cxcl10* and *ifit1* [13]–[16]. Nod2 has been shown to induce many of these inflammatory mediators in different model systems [17]–[19]. The Nod2 receptor has previously been shown to play a role in inducing inflammation in other forms of murine arthritis, but again, its role in *B. burgdorferi* induced arthritis is unknown [20].

Nod2 is a member of the nucleotide-binding domain (NBD) and leucine-rich repeat (LRR) or NLR family of cytoplasmic, pathogen-recognition molecules that is involved in host innate immune defenses [17], [19]. Nod2 recognizes moieties of bacterial peptidoglycan, specifically muramyl dipeptide (MDP), and signals for the activation of pro-inflammatory cytokines [21], [22]. In animal models, Nod2 has been shown to be required for the development of acute, peptidoglycan-induced arthritis [20], and has been shown to contribute to the inflammatory response induced by a number of bacterial pathogens [12], [17], [23]–[26]. Mutations in human Nod2 have also been linked to the development of Crohn's disease, a chronic inflammatory bowel disease [19], [27], [28]. In this latter case, it is counterintuitive that loss of function mutations in a molecule that activates pro-inflammatory pathways can result in the increased inflammatory conditions seen with Crohn's disease. While a number of hypotheses exist to try to explain this dichotomy, there is still no consensus as to the role of Nod2 in different inflammatory diseases [17], [29]–[31]. One possible unifying explanation is that Nod2 may serve different functions in acute versus chronic infections [32] or in infections with a single agent versus conditions where multiple factors, genetic and microbial, are present.

Here, we report on our studies of NLR receptors in *B. burgdorferi* infection. We will show that, similar to prior reports, NLR receptors are important for mediating the inflammatory response to *B. burgdorferi* in acute *in vitro* models. However, we find that *in vivo*, Nod2 deficient mice demonstrate the opposite phenotype, where loss of Nod2 results in increased rather than decreased arthritis. We will present data to explain the discrepancies that are seen between *in vitro* and *in vivo* models of Nod2 involvement in response to *B. burgdorferi* and propose a model that integrates our findings into the understanding of Nod2 function in other systems.

## Materials and Methods

### Ethics Statement

Animals used in *B. burgdorferi* infection experiments encounter minimal discomfort. They are anesthetized with ketamine/xylazine during needle injections to ensure proper subcutaneous injection rather than to relieve pain or discomfort from the injection. The mice developed arthritis, which can be painful and cannot be relieved due to the nature of our studies that focus on the development of arthritis. The arthritis that develops does not impair the animal's ability to feed or drink and peaks at 4 weeks and is resolved by 8 weeks. Tufts Medical Center has a fully approved animal care facility staffed by full-time veterinarians. Any mouse experiencing distress is identified and, if distress cannot be relieved, euthanized. The procedure for euthanasia is consistent with the recommendations of the Panel on Euthanasia of the American Veterinary Medical Association and approved by the Tufts Medical Center Institutional Animal Care and Use Committee. This study was carried out in strict accordance with the U.S. Public Health Service policy and was approved by the Tufts University and Tufts Medical Center Institutional Animal Care and Use Committee (permit number B2010-10). Tufts University and Tufts Medical Center have an Animal Welfare Assurance on file with the Office of Laboratory Animal Welfare at the National Institutes of Health (assurance number A3775-01).

### Mouse and Bacterial Strains

C57BL/6 wild type, TLR2 and Nod2 deficient mice were purchased from Jackson Laboratory. Fully backcrossed Nod1 deficient mice were re-derived in specific pathogen free conditions at Taconic Farms.

Clonal isolates of an infectious, low passage *Borrelia burgdorferi* sensu stricto (strain N40, clone D10E9) were used for all experiments. *B. burgdorferi* was cultured in Barbour-Stoenner-Kelley (BSK)-H medium (Sigma, St. Louis, MO) at 37°C as previously described [33].

### Ligands and Inhibitors

The Nod2 ligand MDP-LD (N-acetylmuramyl-L-alanyl-D-isoglutamine) was purchased from Invitrogen and resuspended to a concentration of 100 mg/ml in endotoxin-free water. ATP (5 mM) was purchased from Invitrogen. Inhibition of endosomal acidification was achieved using Concanamycin A (100 ng/ml), an inhibitor of the V-ATPase, or by monensin (1 µM), an ionophore antibiotic which acts as a Na+/H+ antiporter; both were purchased from Sigma and used at previously published concentrations [10], [18]. These were added to cell cultures 30 minutes prior to stimulation with *B. burgdorferi* [18].

### Generation of Bone Marrow Derived Macrophages

Mouse bone marrow derived macrophages (BMDM) were generated as previously described [7]. Bone marrow cells were flushed from mouse femurs and tibiae with sterile Dulbecco's Modified Eagle Medium (DMEM) and cultured on 100 mm X15 mm plastic petri dishes for 5–7 days in DMEM media supplemented with 30% L929 cell conditioned medium, 20% fetal bovine serum (FBS) and 1% penicillin-streptomycin.

### Infection of Bone Marrow Derived Macrophage Cultures

*B. burgdorferi* were washed three times with DMEM with 10% FBS, counted and resuspended in the same media. Media from confluent cultures of BMDM was removed and replaced with the same media containing *B. burgdorferi* at various multiplicities of infection (MOIs). Cells were harvested at various time points by collecting the cell culture supernatant and then adding TRIzol (Invitrogen) to the remaining cells.

### Activation of Bone Marrow Derived Macrophages

BMDM were treated with IFN-γ at 100 ng/ml for 24 hours prior to subsequent stimulations. This was particularly important in experiments with MDP pre-treatment at 100 µg/ml, since exogenous addition of MDP alone does not induce cytokine secretion in BMDM.

### Mouse Infections

Mice were infected with a dose of 10^4^ *B. burgdorferi* by subcutaneous needle inoculation at 3 to 4 days of age. Infections of mice were confirmed by cultures of ear punch biopsies. Biopsies were cultured in BSK-H media containing rifampicin (50 ng/ml) and phosphomycin (100 ng/ml) and were examined for the presence of *B. burgdorferi* by darkfield microscopy as previously described. Mice were sacrificed between 28 to 30 days (4 weeks) following infection.

### ELISA Measurements

Supernatants were collected from BMDM cultures at 6 hours post stimulation. Cytokines were measured by ELISA using the TNF-α, IL-10 (R&D systems), and IL-6 (e-Bioscience) kits following the manufacturer's instructions.

### Real-time Quantitative (qPCR) Analysis

To perform reverse transcriptase (RT)-qPCR analysis of transcripts, BMDM cells were collected 6 hours after stimulation unless otherwise indicated. RNA was extracted using TRIzol following the manufacturer's instructions. RNA was resuspended in water containing RNaseOut (Invitrogen) and contaminating DNA was removed using the Turbo DNA-free kit (Ambion). Quality of the RNA was checked by examination of RNA integrity on formamide gels. cDNA was synthesized from 1 µg of RNA measured by spectrophotometer using the ImPromII kit (Promega) following the manufacturer's instructions. DNA contamination within the RNA template sample was checked either by use of no RT controls or using primers spanning introns. For measurements of *B. burgdorferi* DNA in tissues, bladders and hearts were collected, and total DNA was extracted using the DNeasy Blood and Tissue Kit (Qiagen).

Quantification of target genes from cDNA or total DNA was performed by qPCR (iCycler, BioRad) using the iQ SYBR Green Supermix 2X (Biorad). Each primer was added to a final concentration to 0.4 nM. qPCR analysis was conducted using the iCycler machine from Biorad. Cycling parameters were 95°C for 15 minutes, 40 cycles of 95°C for 30 seconds, 60°C for 30 seconds, and 72°C for 30 seconds, followed by 95°C for 1 minute, 55°C for 1 minute. Melt curve analysis for purity was performed on each sample by performing 80 cycles of increasing temperature for 10 seconds each beginning at 55°C. Specificity of amplicons was checked by melt curve analysis and gel electrophoresis. For analysis of *recA*, *ospA*, and *nidogen*, the 60°C annealing step was increased to 62°C. Primers used in qPCR analysis of murine *tnf-α*, *il-6*, *il-1β, il-10, ifn-*α universal primers, *cxcl10, ifit1, igtp, mBD4, mBD14, β-actin*, *nidogen*, and the *Borrelia recA* were published previously and/or are listed in Table 1. All samples for each biological replicate were run in duplicates and checked for intra-run variation and, if needed, analyzed again. Whenever possible, different genes/transcripts were analyzed in the same run to avoid inter-run variations. β-actin was analyzed for every run for all samples tested, as a positive control and inter-run calibrator. The number of *ospA*, *recA* and *nidogen* gene copies was quantified using standard curves created using qPCR products amplified from *B. burgdorferi* and murine genomic DNA. Cytokine and β-actin gene expression was normalized from cDNA using the ^ΔΔ^C_t_ method, where the amount of target, normalized to an endogenous reference and relative to a calibrator, is given by 2^−ΔΔCt^, where C_t_ is the cycle number of the detection threshold. All analyses and calculations were performed using the iCycler software.

**Table 1:** Primer sequences used in qPCR analysis.

Primer	Sequence	Reference
*tnf-α*	F: 5′-ATGAGCACAGAAAGCATGATC-3′	[16]
	R: 5′-TACAGGCTTGTCACTCGAATT-3′	
*il-6*	F: 5′-GACTTCACAGAGGATACCAC-3′	[7]
	R: 5′-TATCCAGTTTGGTAGCATCC-3′	
*il-1β*	F: 5′-CAGGATGAGGACATGAGCACC-3′	[7]
	R: 5′-CTCTGCAGACTCAAACTCCAC-3′	
*il-10*	F: 5′-AGAGCTGCGGACTGCCTTCA-3′	[16]
	R: 5′-AATGCTCCTTGATTTCTGGG-3′	
*ifn-α* (universal)	F: 5′- ATGGCTAGRCTCTGTGCTTTCCT-3′	[69]
	R: 5′-AGGGCTCTCCAGAYTTCTGCTCTG-3′	
*cxcl10*	F: 5′- GAAATCATCCCTGCGAGCCTATCC-3′	[13], [14]
	R: 5′-GCAATTAGGACTAGCCATCCACTGGG-3′	
*ifit1*	F: 5′- GTCAACTGTGAGTGCTTCCATCC-3′	[13], [14]
	R: 5′- TCAGGGCAGAAAAGTCAAGGC-3′	
*igtp*	F: 5′-TAGAGCAGACCCACAGAGTTCAGG-3′	[13], [14]
	R: 5′-CAGCAGTCATAGATTTAGACCACGG-3′	
*mBD4*	F: 5′- AACATGCATGACCAATGGAG-3′	[70]
	R: 5′- TCATCTTGCTGGTTCTTCATCT-3′	
*mBD14*	F: 5′- GTATTCCTCATCTTGTTCTTGG-3′	[51]
	R: 5′- AAGTACAGCACACCGGCCAC-3′	
*β-actin*	F: 5′- GTGCGTGACATCAAAGAGAAGC-3′	[16]
	R: 5′- GATGCCACAGGATTCCATACCC-3′	
*recA*	F: 5′-GTGGATCTATTGTATTAGATGAGGCTCTCG-3′	[15], [16]
	R: 5′- GCCAAAGTTCTGCAACATTAACACCTAAAG-3′	
*ospA*	F: 5′- GCAACAGTAGACAAGCTTGAGC-3′	
	R: 5′- GTGTGGTTTGACCTAGATCGTCA-3′	
*nidogen*	F: 5′-CCAGCCACAGAATACCATCC-3′	[15]
	R: 5′-GGACATACTCTGCTGCCATC-3′	

### Quantification of Inflammation

Following euthanasia, each ankle was measured 3 times, and the average of each measurement was determined. For histological staining, tissues were fixed in Bouin's solution for 1 week, and then the hind left leg was skinned, cut in half longitudinally, decalcified, and embedded in paraffin. Sections were then obtained and the slides were stained with hematoxylin and eosin (H&E) by the Rodent Histopathology Core at Harvard Medical School. The slides were then blindly scored for inflammation on a scale of 1 to 3, with a score of 1 representing the least amount of inflammation and a score of 3 representing the greatest amount of inflammation.

### Statistics

For ELISA and qPCR, the mean percentage of cytokine expression relative to the control is reported, with statistical significance determined by Mann-Whitney U analysis. Studies of inflammation in mouse tissue for which data is reported in discrete increments were analyzed by Fisher's exact test. Differences in mouse joint swelling were analyzed by the Mann-Whitney U test.

## Results

### Intracellular Activation of Pro-Inflammatory Cytokines Is Not Solely Dependent on TLR2

**Figure 1:**
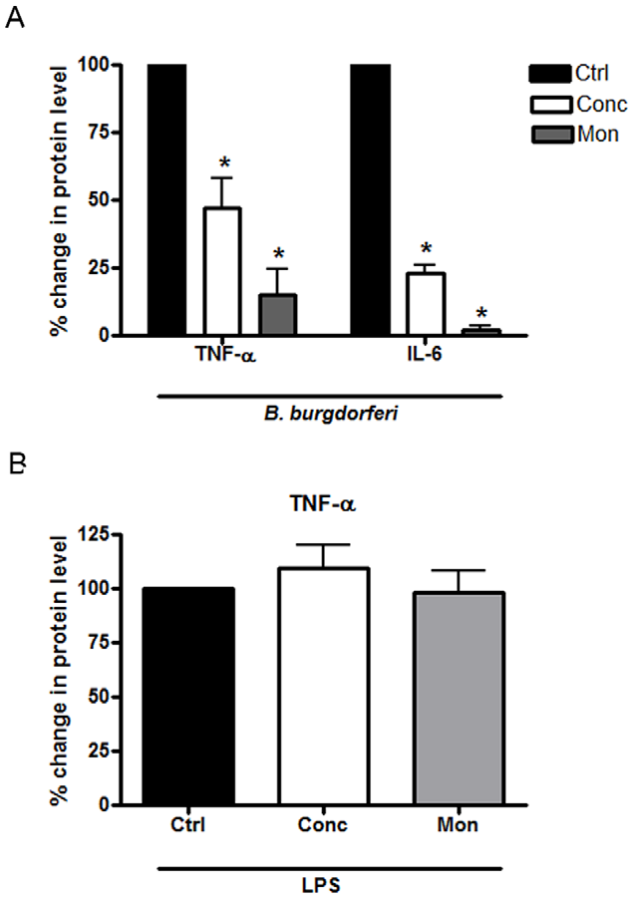
Endosomal processing of *B. burgdorferi* is important for cytokine induction in BMDM. **A**) Murine bone marrow derived macrophages (BMDM) were incubated with *B. burgdorferi* at an MOI 10 for 6 hours and supernatants were collected. Endosomal acidification inhibitors concanamycin A (100 ng/ml) and monensin (1 µM) were added 30 minutes prior to stimulation. Cytokine levels in BMDM supernatants were measured by ELISA. Control cells secreted a mean of 3.95 ng/ml of TNF-α and 3.78 ng/ml of IL-6. Concanamycin treated cells secreted a mean of 1.22 ng/ml of TNF-α and 1.35 ng/ml of IL-6, and monensin treated cells secreted a mean of 1.06 ng/ml of TNF-α and 0.76 ng/ml of IL-6. Bars represent mean percent change in protein levels ± s.e.m. of three independent experiments. Values for control cells (Ctrl) for each experiment were normalized to 100% and other values are shown relative to Ctrl. Because of the normalization, the s.e.m. for Ctrl is zero and no error bars are visible,* p<0.05. **B**) BMDM were stimulated for 6 hours with 5 ng/ml of LPS in the presence or absence of concanamycin A (100 ng/ml) or monensin (1 µM). Supernatants were collected and TNF-α protein was measured by ELISA. Bars represent mean percent change in protein levels ± s.e.m. of three independent experiments. Values for Control cells (Ctrl) for each experiment were normalized to 100% and other values are shown relative to Ctrl. Because of the normalization, the s.e.m. for Ctrl is zero and no error bars are visible, * p<0.05.

It has previously been shown that internalization of *B. burgdorferi* and endosomal acidification are important steps in generating innate immune responses to the pathogen in human macrophages and human macrophage cell lines, as inhibition of endosomal acidification decreased pro-inflammatory cytokine secretion, such as IL-6 secretion [6]–[8]. To confirm that BMDM also require endosomal acidification and maturation to engage innate immune responses to *B. burgdorferi*, prior to stimulation with live *B. burgdorferi*, we treated BMDM with inhibitors of endosomal acidification such as concanamycin A, an inhibitor of the v-ATPase, or monensin, a sodium ionophore which inhibits acidification of the endosome via the Na^+^/K^+^ pump. Cell culture supernatants were harvested after 6 hours of incubation and protein levels of secreted cytokines were determined by ELISA. Concanamycin and monensin decreased induction of IL-6 by 76% and 90% respectively (Figure 1A). In contrast to previously published results of the effects of concanamycin and monensin on TNF-α induction by *B. burgdorferi* in U937 human macrophages, where induction of TNF-α was not significantly dependent on acidification, concanamycin and monensin inhibited TNF-α induction from BMDM by 68% and 80% respectively. To ensure that the effects of the inhibitors were not due to generalized toxic effects, we stimulated BMDM with lipopolysaccharide (LPS) in the presence or absence of the inhibitors. Although the LPS receptor, TLR4, has been shown to signal from the endolysosome for type I interferons, endosomal acidification does not affect LPS-induced secretion of cytokines such as TNF-α, which occurs from TLR4 signaling at the plasma membrane [34]. We found that none of the inhibitors substantially affected TNF-α responses to LPS (Figure 1B), suggesting the inhibitors do not have unexpected non-specific or toxic effects that globally affected cytokine induction.

**Figure 2:**
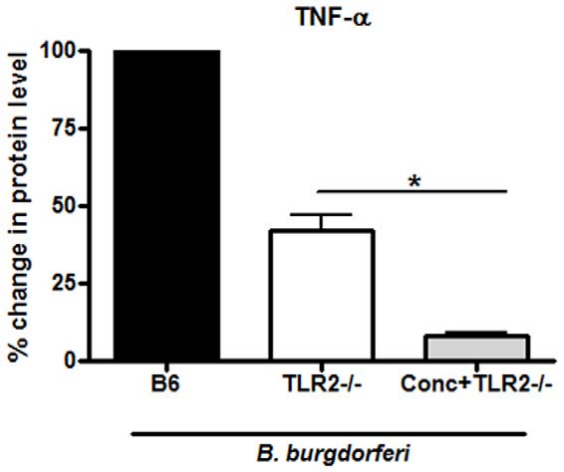
TLR2 independent intracellular signaling plays a role in the host response to *B. burgdorferi* signaling. BMDM from wild type and TLR2 deficient mice were stimulated with *B. burgdorferi* (MOI 10) for 6 hours and supernatants were collected. Concanamycin A (100 ng/ml) was added 30 minutes prior to stimulation. Cytokine levels in supernatants were measured by ELISA. Bars represent mean percent change in protein levels ± s.e.m. of three independent experiments. Wild type cells secreted a mean of 3.51 ng/ml, TLR2 deficient cells a mean of 1.65 ng/ml, and TLR2 deficient concanamycin treated cells a mean of 0.5 ng/ml of TNF-α. Bars represent mean percent change in protein levels ± s.e.m. of three independent experiments. Values for wild type cells (B6) for each experiment were normalized to 100% and other values are shown relative to B6. Because of the normalization, the s.e.m. for B6 is zero and no error bars are visible, * p<0.05.

Having confirmed the requirement for internalization and endosomal maturation in activating murine BMDM responses to *B. burgdorferi*, we next sought to determine whether TLR2 signaling accounts for the entirety of this response. TLR2 is a major innate immune receptor which mediates responses to *B. burgdorferi* and signals from within the phagolysosome in response to *B. burgdorferi* in human macrophages [35]. To determine if TLR2 is the only intracellular signaling molecule that activates pro-inflammatory pathways in BMDM in response to *B. burgdorferi*, we treated TLR2 deficient BMDM with concanamycin A or sham. *B. burgdorferi* (MOI = 10) was then added to the treated cells. ELISAs performed on supernatants collected after 6 hours of incubation showed that the addition of concanamycin A to the TLR2 deficient cells resulted in a further decrease in the secretion of TNF-α by 30% in comparison to TLR2 deficient cells not treated with concanamycin (Figure 2). This suggests that intracellular receptors other than TLR2 also contribute to TNF-α induction in response to *B. burgdorferi*.

### NLRs That Activate Caspase-1 Are Not Activated by the Presence of *B. burgdorferi* in BMDM

**Figure 3:**
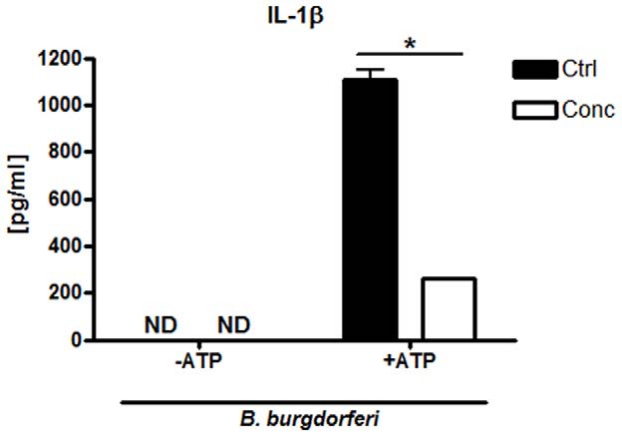
NLRs that activate caspase-1 do not signal in response to *B. burgdorferi* ligands. BMDM were stimulated with *B. burgdorferi* (MOI 10) for 6 hours. In designated samples, 5 mM ATP was added for the last 2 hours of incubation to activate the caspase-1 inflammasome and induce IL-1β secretion. Concanamycin A (100 ng/ml) was added 30 minutes prior to stimulation. Cytokine levels in supernatants were measured by ELISA. Shown is 1 representative experiment from 3 independent experiments. Bars represent the mean secretion of IL-1β in pg/ml ± SD of triplicates in the experiment, *p<0.05.

We were interested in identifying other molecules that could be involved in inducing pro-inflammatory responses from sub-cellular compartments. Candidates included intracellular signaling TLRs such as TLR 7, 8 and 9 and cytosolic receptors from the NLR family. Previous studies have shown that TLR9 does not play a role in the induction of a pro-inflammatory cytokine response in BMDM by *B. burgdorferi* [7]. While TLR7/8 has been shown to activate interferons in response to *B. burgdorferi*, it has not been shown to have a role in activating inflammatory cytokines such as TNF-α [36]. As such, we chose to focus on the NLR family of proteins. While NLR receptors are cytosolic receptors, activation of these receptors is often dependent upon the processing of ligands in endosomes with subsequent sampling of these ligands through poorly understood mechanisms [19]. Of NLRs whose function has been identified, the majority participates in the activation of caspase-1 and cleaves pro-IL1β into mature IL-1β via the inflammasome complex [37], [38]. Stimulating BMDM with *B. burgdorferi* resulted in an increase in IL-1β transcripts (data not shown) [7], but no increase in secreted IL-1β suggesting that the pathways for inducing pro-IL-1β are intact but that *B. burgdorferi* does not directly activate caspase-1 to cleave pro IL-1β (Figure 3). The caspase-1 inflammasome can also be activated by exogenous “danger” signals such as ATP [37], [38], and the addition of ATP with *B. burgdorferi* did result in secretion of IL-1β into cell culture supernatants. In the presence of both *B. burgdorferi* and exogenous ATP, treatment with concanamycin A prior to *B. burgdorferi* stimulation reduced the amount of pro-IL1β transcript and IL-1β secretion, suggesting that signaling and activation of pro-IL1β through other receptors was dependent on endosomal maturation and intracellular signaling (Figure 3). Thus, despite the fact that *B. burgdorferi* expresses flagellin, which is a potential ligand for NLR members such as Ipaf-1 (Nlrc4) and Naip-5 [39]–[43], which activate capase-1 and convert pro-IL-1β into secreted, mature IL-1β, the organism is not able to induce secretion of IL-1β from BMDM in the absence of exogenous ATP. This suggests that *B. burgdorferi* either does not contain the necessary ligands or does not possess the necessary mechanisms to make those ligands available to NLRs that activate the inflammasome, which is consistent with previous observations that caspase-1 is not required for control of murine borreliosis [44].

### Nod2 Is Involved in Transducing Inflammatory Signals Downstream of *B. burgdorferi* in a Model of Acute Infection *in Vitro*

**Figure 4:**
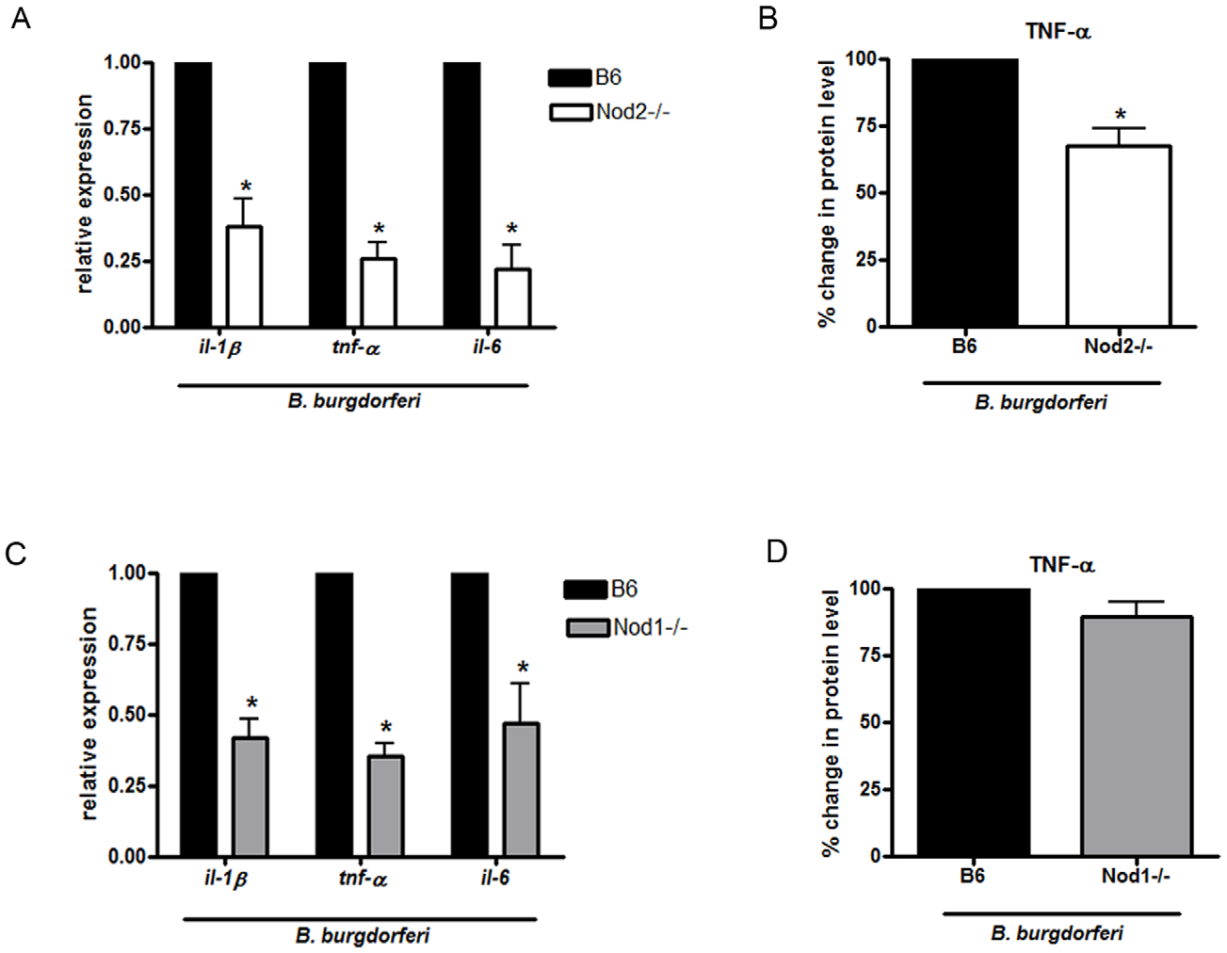
Nod2 but not Nod1 is involved in activating inflammatory responses downstream of *B. burgdorferi*. BMDM from wild type (B6) and Nod2 deficient mice were stimulated with *B. burgdorferi* at MOI 10 for 6 hours. Cytokine expression was measured by RT-qPCR for Nod2 (**A**) and Nod1 (**C**). Values represent mean expression of cytokines relative to control cells ± s.e.m. of three independent experiments. TNF-α protein levels in supernatants were measured by ELISA for Nod2 (**B**) and Nod1 (**D**). For each experiment the control value was set at 100%. Bars represent mean percent secretion relative to control cells ± s.e.m. of three independent experiments. Values for wild type (B6) cells for each experiment were normalized to 100% and other values are shown relative to B6. Because of the normalization, the s.e.m. for B6 is zero and no error bars are visible, * p<0.05. Wild type cells secreted a mean of 3.51 ng/ml of TNF-α and Nod2 deficient cells secreted a mean of 2.35 ng/ml of TNF-α. In Nod1 experiments, wild type cells secreted a mean of 3.95 ng/ml and Nod1 deficient cells secreted a mean of 3.54 ng/ml of TNF-α.

**Figure 5:**
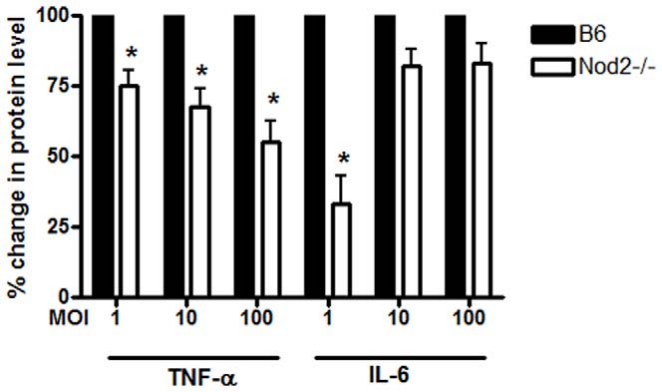
Nod2 mediates the activation of cytokines in response to *B. burgdorferi*. BMDM from wild type (B6) and Nod2 deficient mice were stimulated with *B. burgdorferi* at the designated MOIs for 6 hours. Cytokine levels in supernatants were measures by ELISA; bars represent mean percent change ± s.e.m. of three independent experiments. Values for wild type (B6) cells for each experiment were normalized to 100% and other values are shown relative to B6. Because of the normalization, the s.e.m. for B6 is zero and no error bars are visible, * p<0.05. Raw data for each of the conditions showed that wild type cells secreted a mean of 6.34, 3.51 and 1.8 ng/ml of TNF-α at MOIs of 100, 10 and 1 respectively, while Nod2 deficient cells secreted a mean of 3.52, 2.35 and 1.3 ng/ml at the same MOIs. For IL-6, wild type cells secreted 1.6 ng/ml of IL-6 for MOI 1, while Nod2 deficient cells secreted a mean of 0.58 ng/ml for IL-6 for MOI 1.

NLR family members that activate pathways other than the inflammasome include Nod1 and Nod2, both of which signal through the kinase Rip2 for the activation of NF-κB and MAPKs [19], [38]. To determine whether Nod1 or Nod2 might be involved in responses to *B. burgdorferi*, we stimulated wild type and Rip2 deficient BMDM with *B. burgdorferi*. Rip2 deficient cells showed a 34% decrease in TNF-α secretion by ELISA compared to wild-type cells (data not shown). Because both Nod1 and Nod2 utilize Rip2, we next sought to determine whether either Nod1 or Nod2 (or both) were involved in cellular responses to *B. burgdorferi*. Nod1 and Nod2 deficient macrophages were stimulated with *B. burgdorferi*. Examination of mRNA transcripts for pro-IL1β, IL-6 and TNF-α after 6 hrs of incubation with *B. burgdorferi* showed decreases of 60–75% in Nod1 and Nod2 deficient macrophages compared to wild type (Figure 4). However, when cell culture supernatants were examined by ELISA, only Nod2 deficient cells showed a significant decrease in TNF-α protein levels (Figure 4) or IL-6 protein levels (Figure 5 and data not shown) in comparison with the wild type. Thus, it seems unlikely that Nod1 plays an important role in *B. burgdorferi* signaling, which is consistent with previous observations that Nod1 is not involved in immune response activation downstream of *B. burgdorferi* [12].

We subsequently focused our efforts on understanding Nod2 mediated cytokine responses downstream of *B. burgdorferi*. Nod2 deficient cells consistently showed a decrease in protein levels of TNF-α at different MOIs of *B. burgdorferi* (Figure 5). In contrast, the decrease in IL-6 production was statistically significant only at the lowest MOI tested, MOI = 1, which showed a 65% decrease in Nod2 deficient cells.

**Figure 6:**
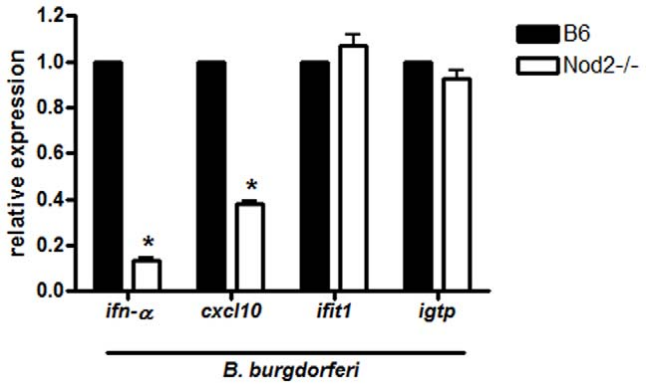
Nod2 mediates the activation of a subset of interferon and interferon responsive genes upon *B. burgdorferi* stimulation. Activated BMDM from wild type (B6) and Nod2 deficient mice were infected with *B. burgdorferi* at MOI 10, cells were washed in DMEM containing 10% FBS and fresh media was added. Cells were collected 16 hours post-infection for *ifn-α* and 6 hours post-infection for *cxcl10*, *ifit1* and *igtp*. mRNA expression was measured by RT-qPCR. Values represent mean expression of cytokines relative to control cells ± s.e.m. of three independent experiments. Values for wild type (B6) cells for each experiment were normalized to 1 and other values are shown relative to B6. Because of the normalization, the s.e.m. for B6 is zero and no error bars are visible, * p<0.05.

We also examined the effects of Nod2 deficiency on induction of type I interferon (IFN) and IFN- responsive genes, which have previously been shown to be important in *B. burgdorferi* infection and are dependent on internalization of the bacterium [9], [13], [14], [36]. Using reverse transcription (RT) quantitative real time PCR-(qPCR), we were able to show that *B. burgdorferi* induction of IFN-α in BMDM was decreased by 85% in Nod2 deficient BMDM when compared to wild-type BMDM (Figure 6).

We were not able to detect activation of IFN-β in response to *B. burgdorferi* and therefore could not address Nod2 involvement in IFN-β activation (data not shown). However, we did observe a Nod2 dependence on *B. burgdorferi* activation of the IFN responsive gene *cxcl10* from BMDM but not the genes *ifit1* and *igtp* (Figure 6). Nod2 deficiency reduced *cxcl10* induction by 60% compared to the wild type.

### Nod2 Deficiency Does Not Have a Significant Effect on Control of *B. burgdorferi* Load *in Vivo*

**Figure 7:**
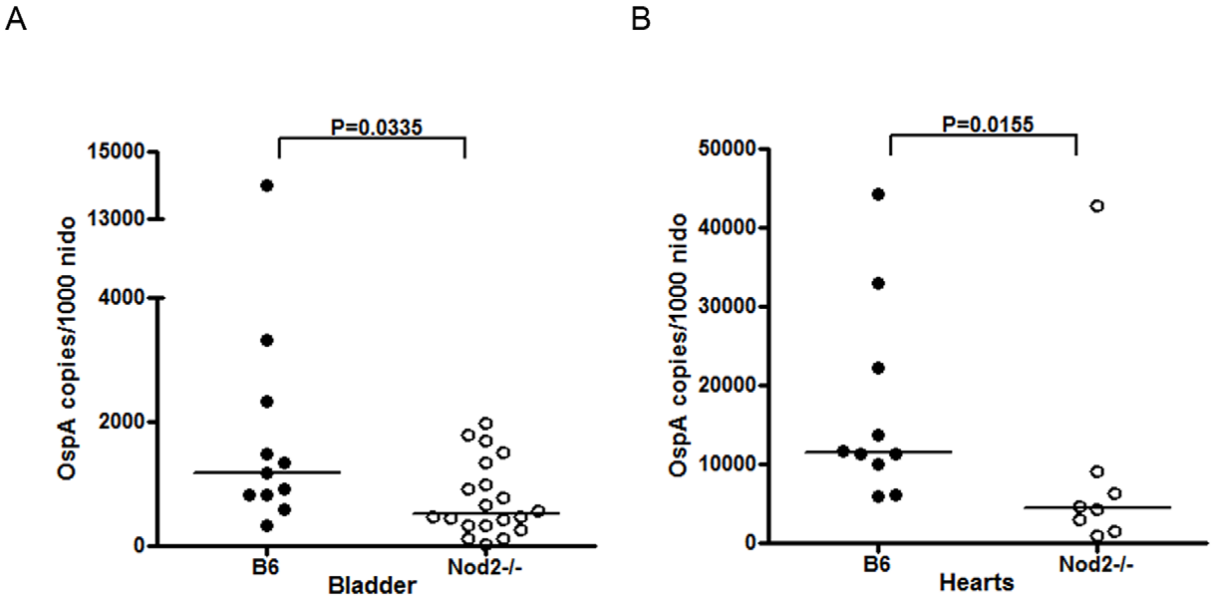
Nod2 deficiency does not affect the control of *B. burgdorferi* burden. **A**) Three or four day old wild type (B6) and Nod2 deficient mice were injected subcutaneously with *B. burgdorferi* at 1×10^4^ cells. Mice were sacrificed at 4 weeks post-infection. Bacterial loads in bladders (**A**) or hearts (**B**) were quantified by quantitative RT-qPCR for the bacterial *ospA* gene and normalized to copies of mouse *nidogen*. Values represent relative expression ± s.e.m. of three independent experiments, * p<0.05.

To address the role of Nod2 in *B. burgdorferi* infection *in vivo*, wild type C57BL/6 and Nod2 deficient mice were subcutaneously infected with the spirochetes. The C57BL/6 genetic background is known to be resistant to *B. burgdorferi* induced arthritis. However, young mice of all species develop more severe arthritis than older mice and even young mice of the C57BL/6 background will develop detectable arthritis. The infant mouse model has been previously used to study other genetic knockout mice on a C57BL/6 background [45]. Age matched wild type and Nod2 deficient mice were infected with *B. burgdorferi* by subcutaneous injection. Mice were sacrificed at 4 weeks post-infection and we examined the numbers of *B. burgdorferi* in heart and bladder tissue by qPCR by measuring copies of *B. burgdorferi recA* (data not shown) and *ospA* (Figure 7) relative to mouse *nidogen.* Despite the immaturity of the immune system at the time of infection, the infection of mice with *B. burgdorferi* at a young age did not affect the bacterial burden in these mice when sampled at 3–4 weeks of age compared to mice infected as adults in previous studies (data not shown) [15]. Our analysis of bacterial burden showed a median decrease from 1190 copies of *ospA* in wild type mouse bladders down to 526 copies in Nod2 deficient bladders and from 11,550 copies in wild type hearts down to 4,475 copies in Nod2 deficient hearts (p = 0.0335 and 0.0155) (Figure 7). This suggests that Nod2 does not play a major role in reducing bacterial burden and may, in fact, hinder control of infection.

### Nod2 Deficiency Leads to Increased Arthritis and Inflammation in Response to *B. burgdorferi* Infection *in Vivo*

**Figure 8:**
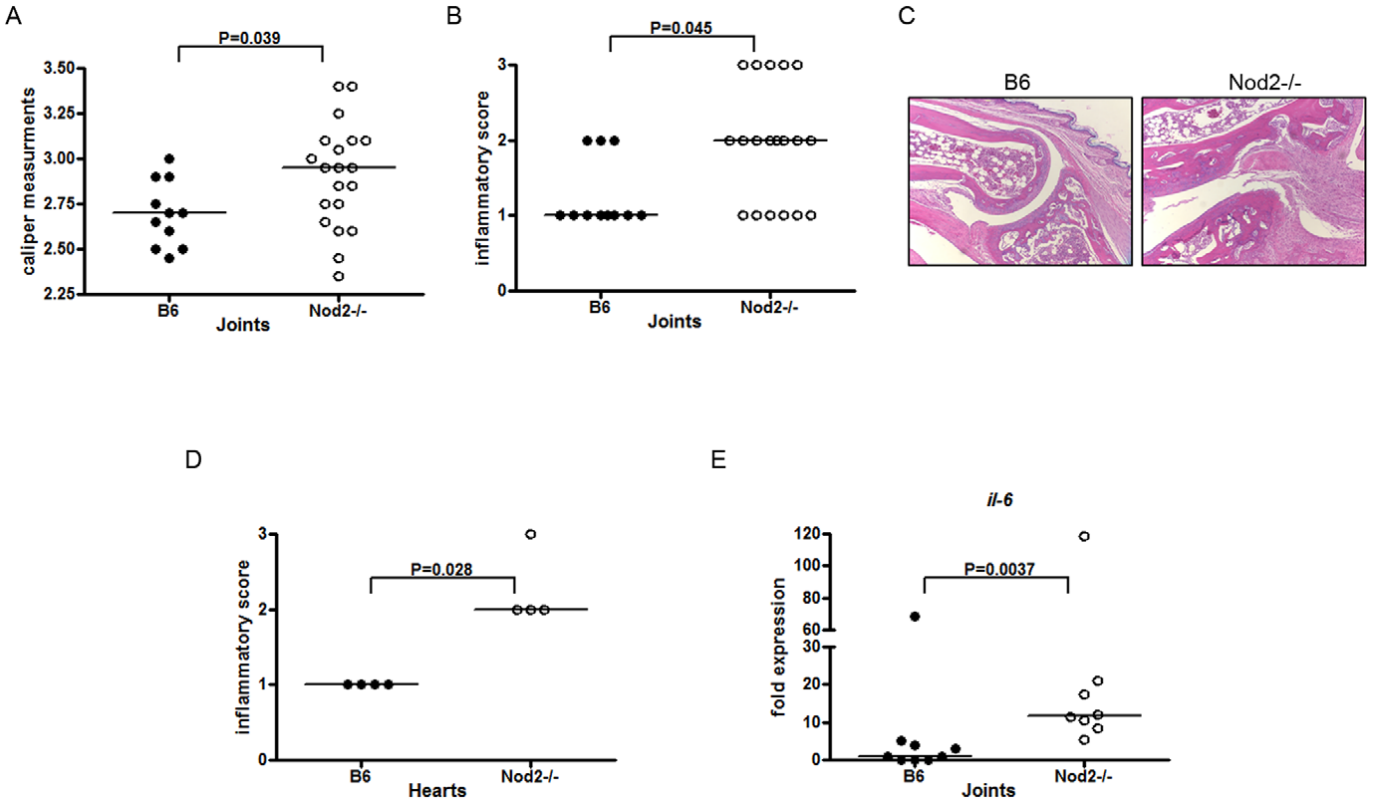
Nod2 deficient mice exhibit increased arthritis and carditis with *B. burgdorferi* infection. **A**) Three or four day old wild type (B6) and Nod2 deficient mice were injected subcutaneously with *B. burgdorferi* at 1×10^4^ cells. Mice were sacrificed at 4 weeks post-infection. Shown are caliper measurements of joint thickness in millimeters at time of sacrifice. **B**) Joints from mice infected in 3 independent experiments were sectioned and stained with H&E and scored blindly by a pathologist for inflammation using a scale of 0-normal, 1- mild, 2-moderate, or 3-severe inflammation. Horizontal lines on the graphs represent median values for the group. **C**) Pictured are representative images of the tibiotarsal joints from an infected wild type and a Nod2 deficient mouse infected with *B. burgdorferi*. In the Nod2 deficient mice, there is severe inflammation with destruction of the cartilage and thickened synovium. The wild type mouse shows minimal inflammation. **D**) Hearts from infected mice were sectioned and stained with H&E and scored blindly by a pathologist with a score of 0-normal, 1- mild, 2-moderate, or 3-severe inflammation. Horizontal lines on the graphs represent median values for the group. Significance was calculated using Fisher's exact test. **E**) Levels of *il-6* transcript were measured by RT-qPCR from joint tissue in wild type and Nod2 deficient mice. The lowest value in the group was arbitrarily set to a value of 1 and all other values are shown in reference to that value using the 2^-ΔΔCt^ method after normalization for the value of *nidogen*.

We determined the effects of Nod2 deficiency on *B. burgdorferi*-induced arthritis and inflammation by characterizing joint swelling in infected mice using both caliper measurements as well as histological scoring by blinded investigators. *B. burgdorferi* infected Nod2 deficient mice showed increased swelling of the tibiotarsal joint compared with wild-type control (p = 0.039) (Figure 8A, B, C). This was consistent with histological grading which revealed significantly increased inflammation in the joints of Nod2 deficient mice infected with *B. burgdorferi* (p = 0.045). An increase in inflammation was further confirmed by measuring the levels of IL-6 mRNA transcripts in infected mouse joints. Mouse *il-6* transcripts were increased by 86% in Nod2 deficient animals compared with wild-type transcripts (p = 0.0127) (Figure 8E). *tnf-α* transcripts were not detectable in the joints of either wild type or Nod2 deficient mice (data not shown).

We also examined the effect of the Nod2 deficiency on carditis in response to *B. burgdorferi*. Nod2 deficient mice showed an increase in inflammation by histological scoring in cardiac tissue (p = 0.028) (Figure 8D). Taken together, these results appear to be inconsistent with *in vitro* models showing a role for Nod2 in inducing inflammatory responses to *B. burgdorferi* and instead suggest that Nod2 may have a role in reducing inflammation.

### Nod2 Does Not Control Inflammation through Induction of IL-10 in a Mouse Model of *B. burgdorferi*-Induced Arthritis

**Figure 9:**
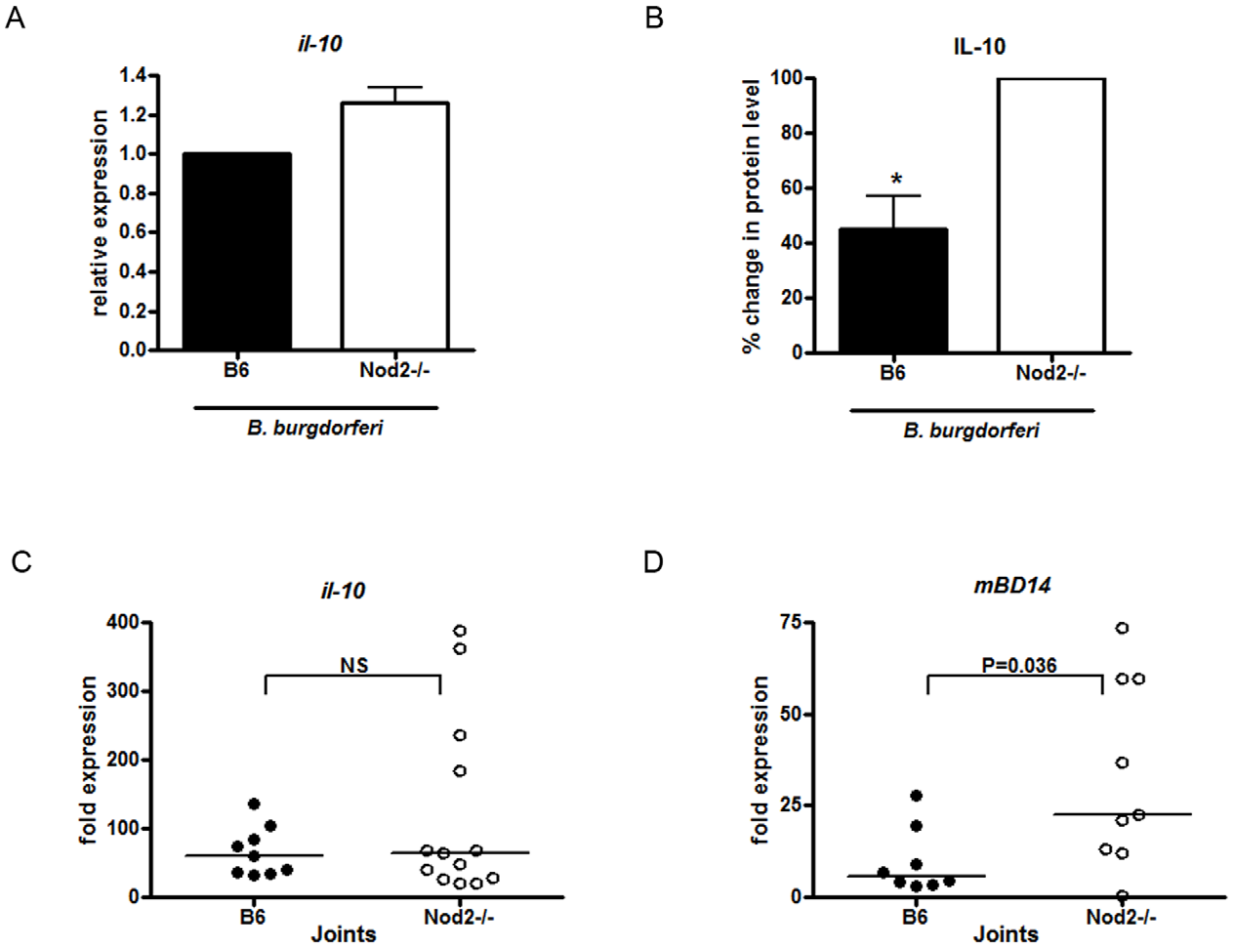
IL-10 and beta-defensin deficiency are not responsible for increased inflammation in joints and hearts of *B. burgdorferi* infected Nod2 deficient mice. **A**) BMDM from wild type (B6) and Nod2 deficient mice were stimulated with *B. burgdorferi* at MOI 10 for 6 hours. mRNA expression for *il-10* was measured by RT-qPCR. Values represent relative expression ± s.e.m. of three independent experiments, * p<0.05. **B**) IL-10 levels in the culture supernatant were measured by ELISA. For each experiment, the control value was set at 100%. Bars represent mean percent secretion relative to control cells ± s.e.m. of three independent experiments. Values for wild type (B6) cells for each experiment were normalized to 100% and other values are shown relative to B6. Because of the normalization, the s.e.m. for B6 is zero and no error bars are visible, * p<0.05. Wild type cells secreted a mean of 80 pg/ml and Nod2 deficient cells a mean of 310 pg/ml. **C**) Three or four day old wild type (B6) and Nod2 deficient mice were subcutaneously injected with *B. burgdorferi* at 1×10^4^ cells. Mice were sacrificed at 4 weeks post-infection and joints were collected and frozen in RNAlater. RNA from joints was isolated by grinding in TRIzol. Transcripts from joints were assessed by RT-qPCR for *il-10* and *mBD14* (**D**). Horizontal lines represent the median values.

We sought to determine the mechanism(s) by which Nod2 signaling could attenuate the inflammatory response to *B. burgdorferi*. Several studies have tried to explain how Nod2 “loss of function” mutations contribute to the development of human Crohn's disease, a chronic inflammatory disorder. Several possible mechanisms have been proposed to explain how loss of Nod2 signaling could result in increased inflammation. One possible mechanism is that Nod2 may be important in the induction of the anti-inflammatory cytokine, IL-10. Some studies have shown that human Crohn's disease associated “loss-of-function” mutations in Nod2 show reduced transcription of IL-10 in response to bacteria [46]–[48]. Pertaining to *B. burgdorferi* specifically, it has also been shown that IL-10 deficient mice exhibit an increase in inflammation of the joints in response to *B. burgdorferi* infection in comparison to wild type mice [49]. However, *in vitro* stimulation of wild type and Nod2 deficient BMDM with *B. burgdorferi* results in a slight increase instead of decrease in *il-10* transcripts (Figure 9A) and secretion (Figure 9B) in Nod2 deficient BMDM compared to wild type cells, suggesting that a direct role of Nod2 on IL-10 transcript and protein secretion does not explain the increase in inflammation in Nod2 deficient mice. Similarly, transcripts for *il-10* in joint tissue from wild-type and Nod2 deficient mice infected with *B. burgdorferi* did not differ significantly (Figure 9C).

### Nod2 May Control Bacterial Load but Not Inflammatory Responses to *B. burgdorferi* through the Induction of Defensins

Another mechanism by which Nod2 has been shown to affect inflammation is through the control of antimicrobial peptide production and reduction of bacterial numbers. Given that *B. burgdorferi* numbers are lower in the Nod2 deficient mice, this did not seem like a likely explanation for the results in our model. However, to confirm this, we examined the effects of the Nod2 deficiency on defensin induction by *B. burgdorferi*. Nod2 has been shown to induce alpha defensins in the intestine, as well as human beta defensin 2 and mouse beta defensin 14 in epithelial cells [17], [50]–[52]. The most common antimicrobial peptides in articular joints include cathelicidin LL37, which is not known to be regulated by Nod2, and human beta defensins 2 and 3 [which correspond to mouse beta defensins (BD) 4 and 14 respectively] [53]. We measured transcripts for murine BD4 and BD14 from inflamed joints. We were not able to detect any induction in *mBD4* in either wild type or Nod2 deficient joints of *B. burgdorferi* infected mice (data not shown). *mBD14* was detectable in arthritic joints of both wild type and Nod2 deficient mice. However, in contrast to studies in the Crohn's model, Nod2 deficient mice showed higher levels of transcription of *mBD14* (5 fold higher) in comparison to wild type mice when infected with *B. burgdorferi* (Figure 9D). Thus, it does not appear that loss of bacterial control due to a decrease in defensins is the mechanism by which Nod2 deficient mice have increased *B. burgdorferi*-induced arthritis.

### Sustained Stimulation of Nod2 Results in the Development of Tolerance to *B. burgdorferi*

**Figure 10:**
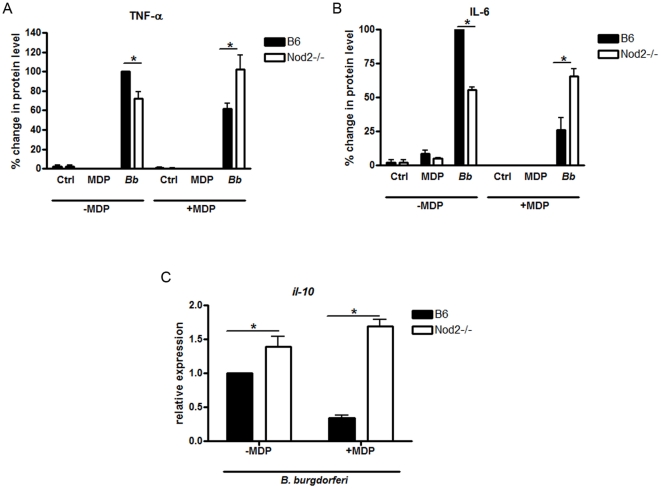
Prolonged activation of Nod2 induces tolerance to *B. burgdorferi*. Activated BMDM from wild type (B6) and Nod2 deficient mice were stimulated with MDP at 100 µg/ml 24 hours prior to stimulation with *B. burgdorferi* at an MOI 10. Just prior to infection, cells were washed in DMEM containing 10% FBS and replaced with fresh media. Supernatants were collected 6 hours post *B. burgdorferi* infection and TNF-α (**A**) and IL-6 (**B**) levels were measured by ELISA. For each experiment, the control value was set at 100%. Bars represent mean percent secretion relative to control cells ± s.e.m. of three independent experiments. Values for wild type (B6), *B. burgdorferi* stimulated cells (*Bb*), in the absence of MDP prestimulation (-MDP), for each experiment were normalized to 100% and other values are shown relative to B6, –MDP, *Bb*. Because of the normalization, the s.e.m. for B6, –MDP, *Bb* is zero and no error bars are visible, * p<0.05. Non-MDP pretreated wild type cells secreted a mean of 420 pg/ml and Nod2 deficient cells secreted a mean of 280 pg/ml of IL-6. MDP pre-treated wild type cells secreted a mean of 40 pg/ml and pre-treated Nod2 deficient cells secreted a mean of 310 pg/ml of IL-6. **C**) *il-10* transcripts were measured by quantitative RT-qPCR, * = p<0.05.

**Figure 11:**
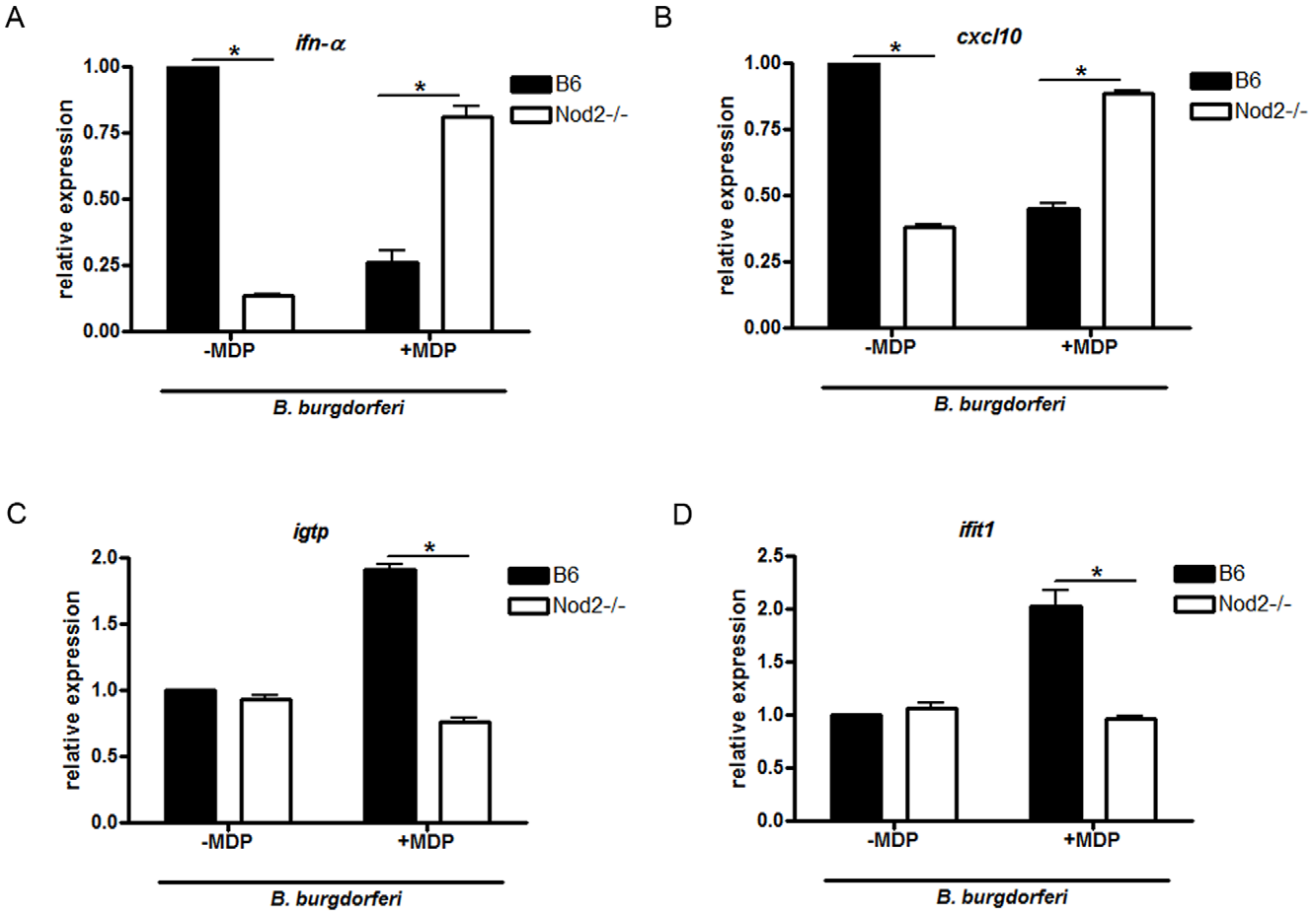
Nod2-mediated tolerance affects *ifn-α*, *cxcl10*, but not *ifit1* and *igtp* mRNA. Activated BMDM from wild type (B6) and Nod2 deficient mice were treated with MDP at 100 µg/ml 24 hours prior to stimulation to *B. burgdorferi* at an MOI 10. Just prior to infection, cells were washed in DMEM containing 10% FBS and fresh media was added. Cells were collected 16 hours post-infection for *inf-α* (**A**) and 6 hours post-infection for *cxcl10* (**B**), *igtp* (**C**) and *ifit1* (**D**)**.** mRNA expression was measured by RT-qPCR. Bars represent mean percent change ± s.e.m. of three independent experiments, * p<0.05.

Previous studies have shown that cells that undergo prolonged stimulation of Nod2, mimicking prolonged infection, no longer respond to subsequent stimulation by bacterial ligands, such as LPS, thus becoming tolerant to those stimuli [32], [54]. We wanted to investigate whether sustained stimulation of Nod2 *in vitro* would decrease the pro-inflammatory response to *B. burgdorferi* through the induction of tolerance. We stimulated activated BMDM with the Nod2 ligand, MDP, at 100 µg/ml or control for 24 hours. We selected this time point because it has previously been established that cells treated with MDP for 24 hours become tolerant to further MDP or TLR stimulus [32]. In our experiments, sustained exposure to MDP resulted in a 40% and 75% decrease in secretion of TNF-α and IL-6 respectively in response to *B. burgdorferi* compared to cells not treated with MDP (p = 0.037, Figure 10A, B). However, in Nod2 deficient cells, prolonged exposure to MDP did not significantly affect levels of protein secretion in response to *B. burgdorferi* when compared to wild type cells. Of note, the levels of *il-10* transcripts in Nod2 deficient cells were elevated in comparison to wild type levels in MDP pre-treated samples, again suggesting that a decrease in IL-10 is not the mechanism for increased inflammation in Nod2 deficient cells (Figure 10C). We also observed greater expression of *ifn-α* and *cxcl10* transcripts by RT-qPCR in Nod2 deficient cells treated with MDP compared with MDP treated wild type cells (Figure 11A, *B*). However, *ifit1* and *igtp* transcripts, which were not affected by Nod2 deficiency (Figure 6), did not show a decrease in expression upon MDP stimulation of wild type cells. Rather, we observed a synergistic effect of *B. burgdorferi* and MDP stimulation on *ifit1* and *igtp* induction in wild type cells that was, as expected, absent in Nod2 deficient cells (Figure 11C, D). These data suggest that prolonged exposure to the Nod2 ligand MDP *in vitro*, results in suppression of pro-inflammatory responses to *B. burgdorferi* and reverses the phenotype seen in cells immediately infected with *B. burgdorferi* where Nod2 appears to play a stimulatory role. The results of these *in vitro* tolerance experiments parallel the IL-6 responses seen in the *in vivo* experiments, where levels are increased in Nod2 deficient mice compared with levels in wild type mice.

## Discussion

Infection with *B. burgdorferi* results in the induction of numerous pro-inflammatory cytokines which are responsible for much of the pathology related to infection. While activation of TLR2 has been shown to account for a majority of the pro-inflammatory response to *B. burgdorferi*, prior reports have also suggested a role for Nod receptors [11], [12]. However, all of the prior reports have studied the role of Nod2 in regulating the immune response to *B. burgdorferi* in *in vitro* models only. Our data with BMDM supports prior reports using microglia, astrocytes and peripheral blood mononuclear cells, showing that the NLR family receptors, and Nod2 specifically, are involved in *in vitro* inflammatory responses to *B. burgdorferi* [11], [12]. We found that Nod2 mediates induction of pro-IL-1β, TNF-α, IL-6, IFN-α and CXCL10 in response to *B. burgdorferi* in BMDM *in vitro*. However, our results with an *in vivo* model of *B. burgdorferi* infection revealed two important findings. First, Nod2 does not appear to play an important role in the control of *B. burgdorferi* infection in mice and in fact, may hinder control. Second, although Nod2 is demonstrably involved in the induction of inflammatory mediators in *in vitro* models of *B. burgdorferi* stimulation, Nod2 appears to have the opposite effect in infected Nod2 mutant mice and is involved in suppressing inflammatory responses during long-term infection through mechanisms of tolerance.

*In vitro*, deletion of Nod2 in murine cells or loss of Nod2 function mutations in the LRR ligand recognition domains of Nod2 from human donors, have been associated with a loss in pro-inflammatory signaling, traditionally measured by a decrease in NF-κB activation, MAPK signaling and pro-inflammatory cytokine secretion [17], [55]. Thus, *in vitro* data from other model systems demonstrating a role for Nod2 has been consistent with prior observations with *B. burgdorferi* [17], [25], [55]–[57].

In other *in vivo* animal models of inflammation where Nod2 deficient mice have been studied, the role of Nod2 deficiency has varied, with some models showing deficient mice having increased inflammation, e.g. ovalbumin antigen-specific induced colitis [58] or *Helicobacter hepatics* induced inflammation [59]), and other models showing deficient mice having decreased inflammation (e.g. the *Salmonella* model of colitis and infection models including *Listeria, Staphylococcus* and *Legionella* [17], [57], [60], [61]). Loss of Nod2 function in *B. burgdorferi* infected mice results in increased, rather than decreased, inflammation in the heart and affected joints as confirmed by both histology and measurement of cytokine expression in joint tissue. Similar discrepancies between *in vitro* and *in vivo* studies have previously been reported in MyD88, CD14 and TLR2 knockout mice [5], [16], [62]–[66]. However, in each of these cases, differences have been attributed, at least in part, to the much higher bacterial burdens (up to 1–2 log more organisms) found in the knockout mice—consistent with the important role of TLRs in controlling bacterial infection. In the case of CD14 deficient mice, an additional role for CD14 in controlling p38 MAPK and negative regulators of inflammation such as suppressors of cytokine signaling (SOCS) have been suggested to mediate inflammation through tolerance [66]. The differences in bacterial burden, although statistically significant, are much smaller in Nod2 knockout mice and are in opposition to what is seen with TLR2, CD14 or MyD88 deficiency in that there is a decrease in bacterial numbers. As such, a role of Nod2 in controlling bacterial burden seems unlikely to explain differences between *in vitro* and *in vivo* studies.

Several hypotheses for mechanisms by which Nod2 deficiency results in increased inflammation in the different systems have been suggested including: a role for Nod2 in the induction of the anti-inflammatory cytokine, IL-10 [48]; a role of Nod2 in the induction of defensins to control bacterial burden [17], [26]; a role of Nod2 in inhibiting processing and secretion of IL-1β [67]; and the role of Nod2 in mediating tolerance by attenuating inflammatory responses initiated by other receptors [32], [54]. We examined each of these mechanisms in our *B. burgdorferi* model. Both *in vitro* and *in vivo* experiments showed no significant effect of Nod2 deficiency on the decrease of IL-10 induction in response to *B. burgdorferi*. Induction of defensins was actually increased in the Nod2 deficient mice, which is consistent with the fact that *B. burgdorferi* numbers were actually lower in Nod2 deficient mice. Finally, in our *in vitro* data using *B. burgdorferi* as a stimulus, we were not able to detect secretion of IL-1β and transcription of pro- IL-1β was reduced in Nod2 deficient cells (Figure 4A,C) rendering it unlikely that Nod2 deficiency leads to increased IL-1β secretion.

The concept that Nod2 may mediate tolerance to stimulation of other receptors such as TLRs has recently been proposed. Administration of the Nod2 synthetic ligand, MDP, attenuates trinitrobenzene sulfonic acid (TNBS) or dextran sodium sulfate (DSS) induced colitis through the down-regulation of several TLR responses [68]. Additionally, Hedl et al. have shown *in vitro* that prior Nod2 activation, mimicking prolonged infection, is capable of inducing tolerance to TLR ligands [32], [54]. Using a similar *in vitro* model as Hedl et al., we were able to show that specific Nod2 stimulation of BMDM with MDP before *B. burgdorferi* infection induces partial tolerance to *B. burgdorferi in vitro*, resulting in the reduction of inflammatory cytokine production and reversing the Nod2 deficiency phenotype seen in acute stimulation of cells with *B. burgdorferi*. In the absence of Nod2, MDP is not able to induce tolerance and Nod2 deficient cells secreted more pro-inflammatory cytokines in response to *B. burgdorferi* than wild type cells. These results correlated with our observations *in vivo* where we found increased inflammatory responses in the joints of Nod2 deficient mice infected with *B. burgdorferi* compared with wild type mice.

It is tempting to hypothesize that the differences seen in the role of Nod2 signaling in the inflammatory response to different infections may be related to the “chronicity” of the infection. In this model, Nod2 is a positive regulator of inflammation during the early stages of infection. However, after a period of sustained stimulation, the role of Nod2 switches and it becomes a negative regulator of inflammation, potentially via the induction of tolerance to further microbial stimulation through either Nod2 itself or other microbial receptors, such as TLRs. Resistance, which clears the invading organisms, and tolerance, which diminishes the negative effects of the host immune response, have been recognized as separate defense strategies in microbial defense. It is possible that molecules such as Nod2 can play roles in both resistance and tolerance during the course of infection. Tolerance may have as important a role for survival of the host as resistance, particularly for prolonged infections such as *B. burgdorferi* where animals are able to co-exist with the bacteria for prolonged periods of time by reducing negative inflammatory reactions. Support for a dual role for Nod2 can be found by comparing acute versus long-term or chronic infection and inflammation in Nod2 deficient mice. Most studies evaluating the role of Nod2 in infection have been done with pathogens that cause acute infection, such as *Listeria*, *Salmonella*, *Staphylococcus* [17], [57], [60], [61]. In these cases, Nod2 deficiency results in decreased pro-inflammatory cytokine levels leading to the reduction of inflammation in response to pathogen infection. Tolerance may not be seen in these infections because the infection resolves or the animal dies after a short period of time. In studies with *Mycobacterium tuberculosis*, a pathogen that establishes long-term infection, Nod2 deficient mice showed decreased pro-inflammatory cytokine production in early stages of infection similar to what is seen with bacteria causing acute infection [23]. However, later in the course of infection, Nod2 deficient mice are more prone to succumb to *M. tuberculosis* infection and die and exhibit increased inflammation in histology sections in comparison to wild type mice [23]. The authors of this study did not propose a mechanism for the late increase in inflammatory responses. In a model where arthritis is induced via a single injection of peptidoglycan, Nod2 deficiency results in decreased inflammation [20]. In contrast, in *B. burgdorferi*-induced arthritis, Nod2 deficiency increases inflammation. While there are numerous differences between these models that could explain why Nod2 could play opposite roles, one obvious difference is in the kinetics of arthritis development. *B. burgdorferi* establishes infection at a distant injection site and then disseminates to the joints and multiplies over weeks to months, providing ongoing stimulus, in contrast to the single injection of peptidoglycan.

In light of recent data showing the role of Nod2 in inducing tolerance to bacterial ligands *in vitro* and the data in this report showing the *in vivo* results of Nod2 deficiency in a slowly progressing arthritis, we believe that a model of differing roles of Nod2 in early and prolonged infection is a likely mechanism to reconcile opposing studies on the role of Nod2 in inflammatory responses. Of note, in murine models of *B. burgdorferi* infection, arthritis eventually resolves despite persistence of the bacteria in the joints. While some of the decrease in inflammation is likely mediated by improved control of bacterial burden over time, host immune systems also utilize mechanisms for regulating inflammation after the initial response in order to limit damage to tissues. Our data supports Nod2 as one of the negative regulators of inflammation in the murine *B. burgdorferi* infection model.

The timing of the change of Nod2 from a stimulatory receptor to an inhibitory receptor in sustained stages of activation and the signaling pathways that control this switch are not known. Some *in vitro* studies have suggested that Nod2 may mediate tolerance by dampening TLR signaling through down regulation of the IRAK-1 kinase and perhaps up regulating the negative regulator of TLR signaling IRAK-M or through negative regulation on TLR signaling mediated by enhanced activation of IRF4 [32], [68]. Recently, a study has shown that Nod2-mediated tolerance is dependent on Nod2 activation of the mTOR pathway and the early secretion of anti-inflammatory mediators IL-10, TGF-β and IL-1Ra [54]. Future studies will be needed to determine the specific pathways involved in control of *B. burgdorferi* mediated arthritis in mice. A more detailed analysis of the regulation of molecules involved in the Nod2 signaling pathway in chronic inflammatory states may reveal important additional molecules and mechanisms that participate in the regulation of signaling during prolonged periods of Nod2 stimulation.
